# The potential impact of invasive woody oil plants on protected areas in China under future climate conditions

**DOI:** 10.1038/s41598-018-19477-w

**Published:** 2018-01-18

**Authors:** Guanghui Dai, Jun Yang, Siran Lu, Conghong Huang, Jing Jin, Peng Jiang, Pengbo Yan

**Affiliations:** 1 Ministry of Education Key Laboratory for Silviculture and Conservation, Beijing Forestry University, Beijing, 100083 China; 2 Ministry of Education Key Laboratory for Earth System Modeling, Department of Earth System Science, Tsinghua University, Beijing, 100084 China; 3 Joint Center for Global Change Studies, Beijing, 100875 China

## Abstract

Biodiesel produced from woody oil plants is considered a green substitute for fossil fuels. However, a potential negative impact of growing woody oil plants on a large scale is the introduction of highly invasive species into susceptible regions. In this study, we examined the potential invasion risk of woody oil plants in China’s protected areas under future climate conditions. We simulated the current and future potential distributions of three invasive woody oil plants, *Jatropha curcas*, *Ricinus communis*, and *Aleurites moluccana*, under two climate change scenarios (RCP2.6 and RCP8.5) up to 2050 using species distribution models. Protected areas in China that will become susceptible to these species were then identified using a spatial overlay analysis. Our results showed that by 2050, 26 and 41 protected areas would be threatened by these invasive woody oil plants under scenarios RCP2.6 and RCP8.5, respectively. A total of 10 unique forest ecosystems and 17 rare plant species could be potentially affected. We recommend that the invasive potential of woody oil plants be fully accounted for when developing forest-based biodiesel, especially around protected areas.

## Introduction

Forest-based bioenergy has attracted worldwide attention due to its potential to mitigate climate change and a number of co-benefits, such as the provision of raw materials, soil and water conservation, and revenue generation in rural areas^1–3^. Among the various types of forest-based bioenergy, biodiesel produced from woody oil plants is especially attractive because it can be used as a green substitute for fossil fuels^4^. Consequently, the production of biodiesel from plantations of woody oil plants is currently under development in Europe^5^, China^6^, and India^7^.

However, the rapid development of bioenergy production may negatively influence biodiversity. The most direct impact is the loss of habitats caused by converting natural ecosystems into plantations^8^. A less visible influence is biological invasion caused by introduced bioenergy plants^9–12^. Many traits that make a plant species suitable for bioenergy production, such as rapid growth, high fecundity, tolerance of a range of climate conditions, and high productivity with low nutrient and water inputs, are the same traits that enable invasive species to successfully spread in new environments^12,13^. There are already examples of woody species planted for bioenergy that have become invasive and have caused significant economic damage. For example, *Prosopis juliflora*, a species initially planted as a feedstock for bioenergy, has become invasive in Kenya. It costs approximately 2,125 USD to clear this plant from one ha of forest stands^14^.

Moreover, global warming is expected to exacerbate biological invasions around the world^15^. Increasing concentrations of atmospheric CO_2_ and global surface temperature as well as altered precipitation patterns will influence plant physiological processes such as photosynthesis and water use efficiency, which can change the growth, development^16^ and plants’ distribution ranges^17^. Evidence already exists of plant species from temperate regions in the Northern Hemisphere shifting poleward^17,18^. These changes have been observed in invasive woody plant species as well. Increased atmospheric CO_2_ concentrations have been found to stimulate the growth and reproductive rates of invasive woody plant species^19^. Studies have shown that the distribution ranges of some invasive woody plants used for bioenergy production might increase in response to climate change. For example, *Triadica sebifera*, an invasive species used as a bioenergy plant in the United States, could spread 200 km northward of its current distribution range under a scenario of a 2 °C temperature rise by 2050, which would pose invasion risks to many ecosystems^20^.

The development of forest-based biodiesel poses a high invasion risk. Because viable biodiesel production needs a stable supply of large amounts of fruits or seeds^21,22^, woody oil plants must be planted on a large scale and maintained for a long time. A woody oil plant species will produce a constant pool of propagules. At the same time, such species will be subjected to the influence of climate change. These factors can potentially contribute to the invasion of a woody oil plant species into new ecosystems if it has invasive traits. Therefore, an assessment of the invasive potential of woody oil plants under current and future climates is urgently needed.

To bridge this knowledge gap, we conducted a case study in China on the invasion risk posed by woody oil plants used for biodiesel production. China has great potential to develop forest-based biodiesel, with 154 woody oil plant species that contain more than 40% fat content in fruits or seeds^23^. In addition, more than 43.75 million ha of marginal lands can potentially be used for growing plantations of woody oil plants^24^. In 2013, the State Forestry Administration (SFA) of China issued the *Development Plan for Forest-based Bioenergy* to guide the development of forest-based bioenergy in China between 2012 and 2020^25^. Once the plan is implemented by 2020, the total area of woody oil plant plantations will increase from 1.35 million ha to 4.22 million ha, and the potential yield of biodiesel will reach 580 million tons of standard coal equivalent. At the same time, China is predicted to experience dramatic climate changes. The annual mean air temperature is projected to increase by 2 to 3.7 °C under RCP8.5 during the time period 2041–2069, while precipitation is projected to increase over a range of 2 to 20%^26^. Due to the large-scale planting activity and predicted climate change, there is a real danger that developing forest-based biodiesel will facilitate the spread of invasive woody plant species into China’s natural ecosystems. To date, this potential risk has not been studied and has been entirely neglected in the policy-making process.

We have two specific objectives in this study: (1) to predict the potential distribution ranges of woody oil plant species that are considered invasive in China under current and future climates and (2) to predict the invasion risks posed by these invasive woody oil plant species to protected areas for plant species or forest ecosystems in China.

## Results

### Invasiveness of woody oil plants in China

Among the 154 woody oil plants that have the potential to be used in the production of biodiesel in China, three are considered invasive: *Jatropha curcas*, *Ricinus communis*, and *Aleurites moluccana*. *J. curcas* has been chosen by the SFA as one of the six species to be planted on a large scale^25^. Currently, *J. curcas* can be found in the tropical and subtropical regions of southern China, including in Hainan, Guangdong, Guangxi and Yunnan Provinces and southern parts of Sichuan, Guizhou and Fujian Provinces. *R. communis* is distributed in southern, southwestern, and central China. *A. moluccana* is mainly distributed in a narrow tropical region in southern China, including Hainan Province, and the southern parts of Guangdong, Guangxi, Yunnan, and Fujian Provinces. The distribution ranges of *A. moluccana* and *J. curcas* overlap in the southern parts of Guangdong, Guangxi, Yunnan, and Fujian Provinces, while those of *J. curcas* and *R. communis* overlap in Yunnan, Guangxi, Guangdong and Hainan Provinces and the southern parts of Sichuan, Guizhou and Fujian Provinces. Among the three species, the invasiveness of *R. communis* is the strongest according to *The Checklist of Chinese Invasive Plants*^27^, followed by *J. curcas* and *A. moluccana*.

### Accuracy of the model results

**Figure 1:**
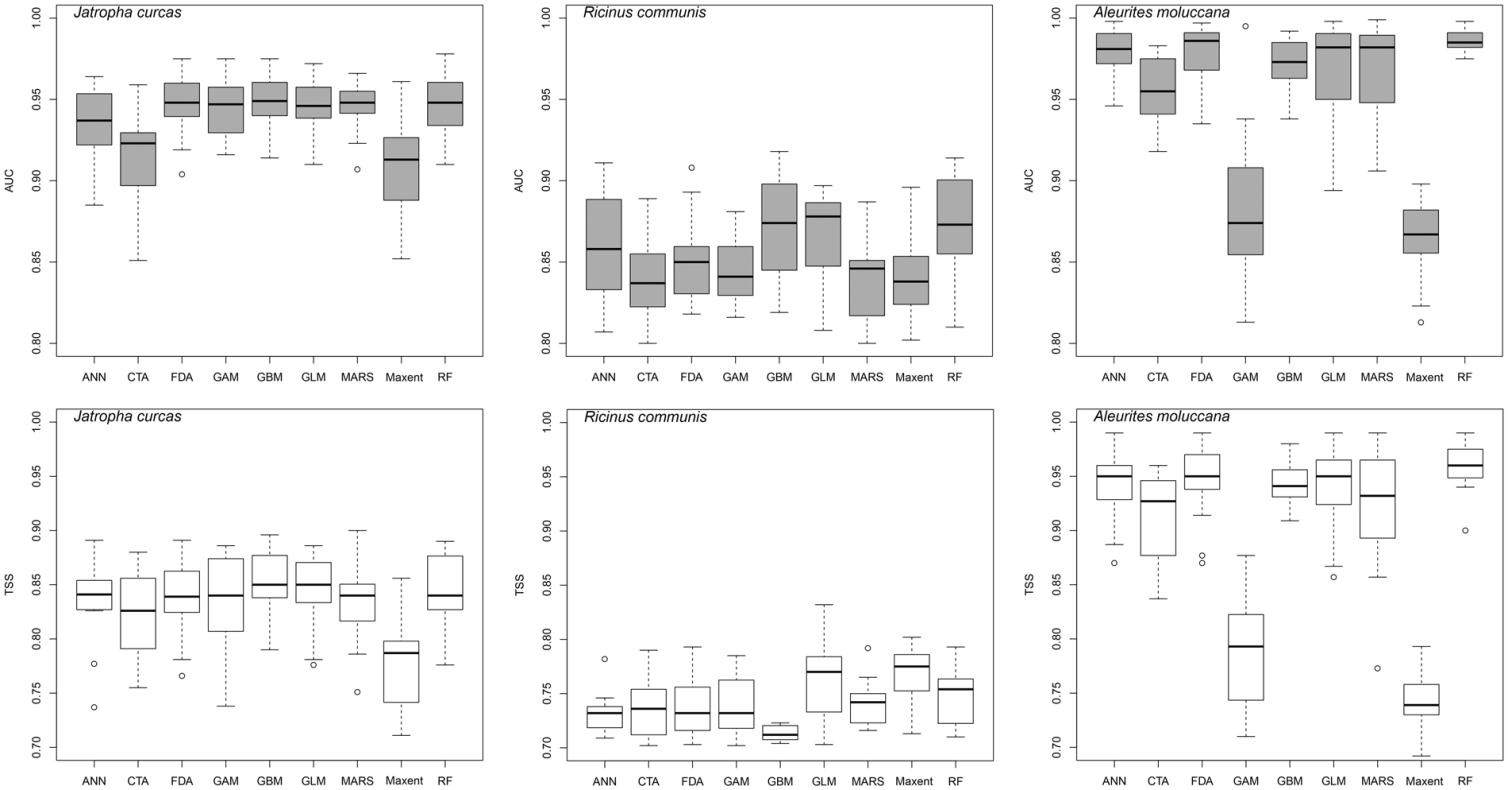
Values of AUC and TSS for modeling outputs. ANN, artificial neural network; CTA, classification tree analysis; FDA, flexible discriminant analysis; GAM, generalized additive model; GBM, generalized boosted regression model; GLM, generalized linear models; MARS, multivariate adaptive regression splines; Maxent, maximum entropy; RF, random forests. The figures were created using *R*^30^ (Version 3.3.2, *R* Core Team, https://www.r-project.org/).

The accuracy of the modeling results was examined using area under the receiver-operating characteristic curve (AUC) values and true skill statistics (TSS). All model outputs had a mean AUC value greater than 0.8 and a mean TSS value greater than 0.7 (Fig. 1), which are considered acceptable for SDM results^28,29^.

### Range shifts of the three species under future climates

**Figure 2:**
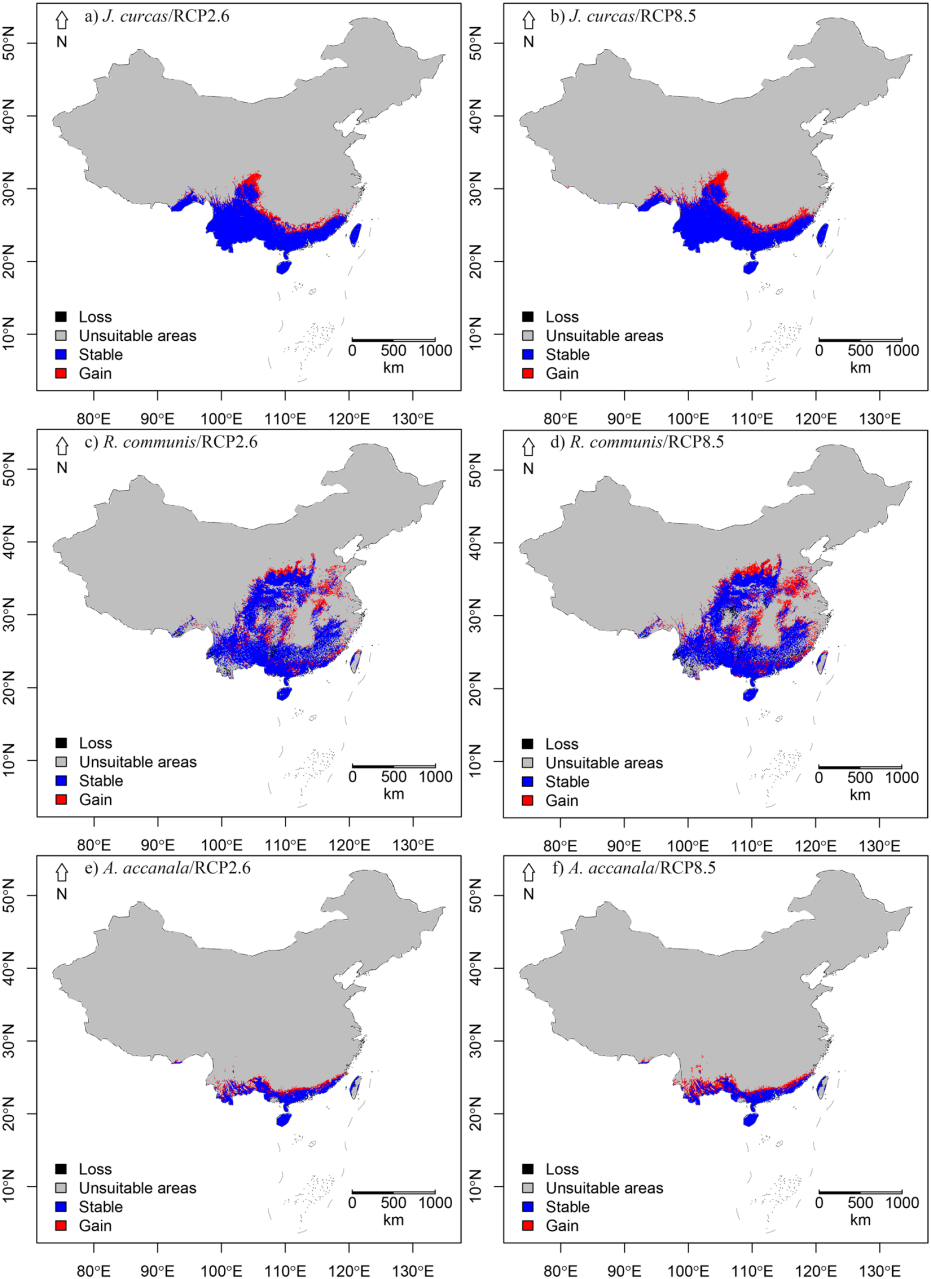
The current and future suitable habitats of the three species in China by 2050: (**a**,**b**) distribution ranges of *J. curcas;* (**c**,**d**) distribution ranges *of R. communis*; (**e**,**f**) distributions of *A. moluccana*. Blue-colored areas are currently suitable habitats that may still be suitable in the future. Red-colored areas are suitable habitats added in the future. Black-colored areas are currently suitable habitats that become unsuitable in the future. The figure was created using *R*^30^ (Version 3.3.2, *R* Core Team, https://www.r-project.org/) and the *R* package *mapdata*^31^. The base map of China was from *mapdata*.

The results show that the suitable habitats of the three invasive species may expand from 15% to 50% under future climate conditions (Fig. 2). The changes are mainly caused by northward expansion.

For all three species, expansion was greater under the high emission scenario. The largest percentage of increase (50%) was predicted for *A. moluccana* under the RCP8.5 Scenario (Table 1). *R. communis* had the largest expansion of area, which was 561,384 km^2^. The overlaps of the potentially suitable areas between their current and 2050 values exceeded 95% for all three species.

**Table 1:** Potential changes in suitable habitats for three invasive woody oil plant species in China by 2050.

**Species**	**Current (km** ^**2**^ **)**	**RCP2.6**				**RCP8.5**			
		Loss (km^2^)	Stable* (km^2^)	Gain (km^2^)	Net (%)	Loss (km^2^)	Stable (km^2^)	Gain (km^2^)	Net (%)
*Jatropha curcas*	1,269,140	368	1,268,772	192,280	+15	276	1,268,864	278,116	+22
*Aleurites moluccana*	340,768	5704	335,064	117,392	+33	3772	336,996	175,812	+50
*Ricinus communis*	1,680,620	73,784	1,606,872	393,024	+19	89,240	1,591,416	561,384	+28

^*^“Stable” means that habitats are suitable now and may still be suitable for the studied species by 2050. “Gain” indicates new suitable habitats added in the future. “Loss” means that habitats suitable now but may become unsuitable by 2050.

### Invasion risks posed by the three species to protected areas

The overlaps between the future suitable habitats of the three species and protected areas indicated the existence of invasion risks (Table 2). The invasion risk posed by *A. moluccana* was the lowest. Four protected areas and a total of 368 km^2^ of protected area may be invaded by the species under the low emission scenario (Table 2). The invasion risk posed by *R. communis* was the highest, and the species may invade 19 protected areas and 2070 km^2^ of protected areas under the high emission scenario. In total, 42 protected areas are potentially at risk. One protected area may be threatened by two species, and 41 protected areas are threatened by one species. Among them, 12 reserves are designated to protect plant species, and the rest are set up to protect specific forest ecosystems. The potential risk was greater under the high emission scenario. A total of 26 reserves will be at risk under RCP2.6, while the number reaches 41 under RCP8.5 (detailed information regarding these protected areas can be found in supplementary Table S1).

**Table 2:** The number of protected areas and the total area of lands that may be threatened by the three species in China by 2050.

Species		*Jatropha curcas*	*Aleurites moluccana*	*Ricinus communis*
RCP2.6	Number of reserves	11	4	11
	Number of affected plant species	7	0	7
	Number of affected forest ecosystem	3	3	6
	Area (km^2^)	2329	368	1288
RCP8.5	Number of reserves	14	8	19
	Number of affected plant species	8	2	8
	Number of affected forest ecosystem	3	4	12
	Area (km^2^)	3312	920	2070

The protected areas that may be impacted by *A. moluccana* are mainly concentrated in a narrow region in southern China, including the southern parts of Guangdong, Yunnan, Guizhou, and Sichuan Provinces. The protected areas that may be invaded by *J. curcas* are distributed to the north of the areas affected by *A. moluccana*, including the northern parts of Guangdong, Guizhou, and Sichuan Provinces and the southern parts of Fujian, Jiangxi and Chongqing Provinces. *R. communis* may affect areas in southwestern and central China. Some protected areas may be affected by more than one species. For example, the Chishuisuoluo Natural Reserve in Guizhou has been established to protect *Alsophila spinulosa* (Wall. ex Hook.) R.M. Tryon, and the reserve may be invaded by both *J. curcas* and *R. communis*.

The three species may pose invasive risks to 12 protected areas that are protecting rare and endangered plant species. For example, the Liangyeshan Natural Reserve in Fujian province was established to protect *Taxus chinensis*, *Bretschneidera sinensis* and *Ginkgo biloba*, which are listed as endangered (EN) on the IUCN Red List. The three invasive woody oil plant species also may pose risks to 30 nature reserves that have been established to protect specific forest ecosystems. For instance, *R. communis* may potentially invade the Shennongjia Natural Reserve, Houhe Natural Reserve, and Mulinzi Natural Reserve, which have been established to protect the mid-subtropical forest ecosystem in Hubei province. The Fengxi Natural Reserve and Qimuzhang Natural Reserve, which have been established to protect subtropical evergreen broad-leaved forest ecosystems in Guangdong province, may be threatened by *A. moluccana*.

## Discussion

Our results show that the suitable habitats of the three woody oil plant species may shift to the north or northwest under future climate conditions. Our finding that climate change will impact the suitable planting areas of the three species is in line with those of similar studies. For example, the distribution ranges of *S. sebiferum* in the United States^20^, *Olea europaea* in the Mediterranean basin^32^, and *Millettia pinnata* in Australia^33^ have all been predicted to increase under future climate conditions. The suitable habitats of oil palm may shrink in Malaysia and Indonesia and even globally due to climate change^34,35^. One possible reason for the projection of the northward expansion of distribution ranges of the three species in the present study is their origins. These species are derived from either tropical or subtropical regions^36–38^. It is projected that the future climate in China will become warmer. Many areas classified as unsuitable habitats for these species under the current climate may become suitable as temperatures increase.

However, our results contrast with those of early studies that predicted that climate change may have little impact on the suitable habitat of *J*. *curcas* in China^39^ or may even have a negative impact^40^. The discrepancy may be caused by the different species occurrence data, climate inputs, and modeling approaches used, which can result in variations in the direction and magnitude of range changes^41–44^. In comparison with the earlier study, we used the latest climate projections (outputs of CMIP5) and three times more species occurrence records. In addition, we used an ensemble model, while the early study used a single model (e.g., domain) to predict the suitable habitat of *J. curcas*. Compared to the single-model approach, the ensemble-model approach has been proven useful for reducing potential bias and increasing confidence in predictions^45–48^.

The potential invasion of *J. curcas* into protected areas can be detrimental to the conservation of endangered species and specific types of forest ecosystems. Studies have shown that *J. curcas* can alter the nutrient cycling process and increase risks of ecotoxicity and water depletion^49,50^. Because of its drought-tolerant character, *J. curcas* is expected to develop a higher biomass under a warmer and drier climate and outcompete endangered plants for light, moisture and space. Similarly, invasions by *R. communis* and *A. moluccana* may cause significant biodiversity losses and changes in ecosystem functions in protected areas, as extensive studies have shown that invasive plants can affect functionality and biodiversity at the ecosystem level^8,51–53^.

Evidence suggests that large-scale plantings of species with traits that enhance rapid spread and survival can increase the invasion risks to susceptible ecosystems^9–11^. Our findings suggest that the forest-based biodiesel industry should heed this warning. As exemplified in our study, protected areas in China could be threatened when future climate conditions provide suitable habitats for the three invasive woody oil plant species. These results can provide managers with a better understanding of the areas to which each of the three invasive oil plants has a high probability of spreading and can be used to allow more thorough early detection and rapid response.

Based on our results, we recommend that forestry administrations in China proceed carefully when planting invasive woody oil plant species. Among the three species, *J. curcas* has been selected by the SFA as one of six species to produce biodiesel. SFA plans to grow 1.45 million ha of new plantations of *J. curcas* by 2020^25^, but caution should be exercised when planting this species. In particular, planting this species near protected areas that are suitable habitats in current and future climate conditions should be avoided. Other measures, such as creating buffer zones around plantations and having a long-term invasive species management plan in place, can also help reduce the invasion risk posed by *J. curcas*.

The limitations of our study should be noted. For example, we assumed a full dispersal scenario for all three species. Factors such as land use^54^ and ecological interactions among species^55^ that are crucial for a species to realize its projected distribution were not considered in our study. The SDM approach itself also contains uncertainty^56^. Another limitation is that certain areas may be currently suitable but not occupied and will remain suitable in the future. However, because there are no data on the true absence of a species in protected areas, it is impossible to single out such areas using presence data only. Despite these limitations, our study has for the first time revealed the potential invasive risk posed by woody plants used for biodiesel production to protected areas in China. The information is useful for forestry policy makers in China and in other countries where large-scale forest-based biodiesel production is under development.

## Methods

### Assessment of invasiveness

To assess the invasiveness of species, we looked up the 154 woody oil plants (see names in Supplementary Table S2) in the *Global Invasive Species Database* (GISD)^57^ and *The Checklist of Chinese Invasive Plants*^27^. Only species appearing in both databases were considered to be invasive in China.

### Data

Occurrences of the three invasive woody oil plants were compiled from the Chinese Virtual Herbarium (CVH)^58^ and Plant Photo Bank of China (PPBC)^59^. The compiled occurrence data include two types: (1) records with names of places and geodetic coordinates and (2) records with names of places but no geodetic coordinates. For the second type of records, the center coordinates of the smallest administrative unit corresponding to the name of the place was used as the coordinates of the occurrence record. A total of 813, 492, and 278 records were compiled for *J. curcas*, *R. communis*, and *A. moluccana*, respectively. A spatial filtering approach was then used to reduce spatial bias by merging all occurrence records in the same grid (size = 10 km) as one occurrence. After spatial filtering, 343 records of *J. curcas*, 135 records of *R. communis*, and 67 records of *A. moluccana* remained.

We downloaded 19 bioclimatic variables from WorldClim^60^. The observed data from 1950 to 2000 represent the base climate. Averaged values of bioclimatic variables constructed from climate projections (2050 s) of 18 GCMs (the names of the GCMs are in the Supplementary Table S3) were used as future climates. Two representative concentration pathways, the RCP2.6 low emission scenario and the RCP8.5 high emission scenario, were included. Four biophysical variables—altitude, slope, aspect, and soil classification—were extracted from the Harmonized World Soil Database (webarchive.iiasa.ac.at/Research/LUC/External-World-soil-database/HTML/).

To reduce the collinearity of the variables, we ran a Pearson correlation analysis using 19 bioclimatic variables. When the correlation coefficient between two variables >|0.8|, one of them was removed. We kept the one that was more relevant to the distribution of the species and was easy to interpret. For example, annual precipitation and precipitation in the wettest month were correlated, and we dropped the latter variable because the effect of annual precipitation is easier to interpret. In the end, we kept six bioclimatic variables and four biophysical variables: minimum temperature of the coldest month, isothermality, temperature seasonality, mean temperature of the warmest quarter, annual precipitation, precipitation seasonality, altitude, slope, aspect, and soil classification.

Base maps of protected areas in China were downloaded from the World Database of Protected Areas^61^. We selected 267 protected areas that are designated for the protection of specific plants or specific forest ecosystems. These areas are expected to be impacted by invasive woody oil trees more directly than other types of protected areas^62,63^.

### Modeling process

The ensemble model can reduce both false-negative and false-positive errors in predictions of species distribution^64^. We used an ensemble of ten algorithms included in the *R* package *biomod2*^65^: generalized linear models (GLMs), generalized additive models (GAMs), multivariate adaptive regression splines (MARS), artificial neural networks (ANNs), random forest (RF), generalized boosted regression models (GBMs), maximum entropy (Maxent), classification tree analysis (CTA), flexible discriminant analysis (FDA), and surface range envelope (SRE).

For each species, we generated pseudo-absence data randomly following the method used in Wisz and Guisan^66^. First, we used “surface range envelope (SRE)” to map the potential habitat suitability for the study species. Then, we selected pseudo-absences randomly from the areas outside of the potentially suitable habitat. We compared the influences of different numbers of pseudo-absences in the model outputs. We found that the ensemble result would not be significantly affected, while the results of a single model might vary. Therefore, we used different numbers of randomly generated pseudo-absences for different species. We only generated the same number of pseudo-absences as presence records (i.e., 1000 for *J. curcas*, 500 for *A. moluccana*, and 500 for *R. communis*). This procedure was repeated three times for each species to address potential sample bias in the absences.

We ran the models using their default settings as suggested by Thuiller *et al*.^65^. For each species, the models were run using a random set of 70% of the available data (presences and pseudo-absences) and evaluated against the remaining 30% of the data. The area under the receiver-operating characteristic (AUC)^28^ and true skill statistics (TSS) were used as indicators of model performance^29,47^. The modeling process was replicated five times (five-fold cross validation) for each selected species. AUC values greater than 0.8 indicate good model performance, while values below 0.5 indicate modeling results no better than random. In general, values of TSS < 0.40 are considered poor fits, while values from 0.40 to 0.75 are considered good fits. Those results with values > 0.75 are considered excellent fits^67^. For each species, we generated 135 model runs (3 replicate pseudo-absence datasets × 9 algorithms × 5 replicates) for each time period.

Following the method developed by Engler *et al*.^68^, the final ensemble model prediction was calculated as the weighted average of the individual model outputs and the TSS evaluation scores were used as weights. We only kept models with TSS values above 0.7, as suggested in the literature^69^. Therefore, the SRE model was excluded because the TSS values for all three species were below 0.7. Finally, one ensemble projection of the current suitable habitat and two ensemble projections of future suitable habitat for each species were obtained and converted into maps. The continuous habitat suitability scores were converted to suitable/unsuitable habitats using the maximum TSS method^70^. The full dispersal assumption was used in the simulation, which means that species could expand to any suitable areas in the future. We identified the loss, stable, and newly gained areas for each species by comparing projections of future suitable habitats to their current suitable habitats. “Stable” means that the habitats are suitable now and may remain suitable for the studied species by 2050. “Gain” indicates new suitable habitats added in the future. “Loss” means that habitats are suitable now but may become unsuitable by 2050. The biomod2 results were output to *R* software^30^ (Version 3.3.2, *R* Core Team, Austria) to generate these areas.

### Estimating the invasion risks posed by the species on protected areas

To estimate the invasion risks of the three species to protected areas, we overlaid the distribution ranges of the three species over the base map of 267 protected areas. We considered a protected area to be potentially threatened by an invasive woody oil plant species in a future climate if the current distribution map of the species did not overlap with the protected area but the future distribution map did. These protected areas may become suitable for the species as a result of climate change. The acreages of overlapped areas were then extracted from the map for each species.

## Electronic supplementary material

Supplementary information
