# Supplementary material for: The potential impact of invasive woody oil plants on protected areas in China under future climate conditions
Source: Sci Rep. 2018 Jan 18;8:1041. doi: 10.1038/s41598-018-19477-w (PMC5773687; doi:10.1038/s41598-018-19477-w)
Supplement: Supplementary file 1 — Supplementary information [file 41598_2018_19477_MOESM1_ESM.doc]

***Scientific Reports***

**Supplementary information**

**The potential impact of invasive woody oil plants on protected areas in China under future climate conditions**

Guanghui Dai, Jun Yang^*^, Siran Lu, Conghong Huang, Jing Jin, Peng Jiang, Pengbo Yan

Table S1. The protected areas threatened by the three invasive woody oil plants under future climate conditions

Table S2. The list of 154 woody oil plants

Table S3. General circulation models (GCMs) used in this study

**Table S1. The protected areas threatened by the three invasive woody oil plants under future climate conditions**

| **Species** | **Province** | **Reserves Name** | **Establishing Time** | **Major protected subjects** | **Affected area (km^2^)** | |
| --- | --- | --- | --- | --- | --- | --- |
|  |  |  |  |  | **RCP2.6** | **RCP8.5** |
| *J. curcas* | Fujian | Liangyeshan | 19950101 | *T. chinensis*, *B. sinensis, G. biloba* | 255 | 411 |
|  | Jiangxi | Ganjiangyuan | 20011001 | Mid-subtropical evergreen broad-leaved forest | 85 | 236 |
|  |  | Jinpenshan | 19820331 | Mid-subtropical evergreen broad-leaved forest | 84 | 84 |
|  |  | Jiulianshan | 19810306 | Mid-subtropical evergreen broad-leaved forest | 79 | 83 |
|  | Guangdong | Nanling | 19840401 | Mid-subtropical evergreen broad-leaved forest | 170 | 176 |
|  | Guizhou | Chishuisuoluo | 19840911 | *A. spinulosa* | 250 | 90 |
|  |  | Chishuiyuanshenglin | 19900701 | Mid-subtropical evergreen broad-leaved forest | - | 273 |
|  | Chongqing | Simianshan | 19920401 | Subtropical evergreen broad-leaved forest | 278 | 280 |
|  |  | Jinyunshan | 19790401 | Subtropical evergreen broad-leaved forest | 89 | - |
|  | Sichuan | Huagaoxi | 19980101 | *A. spinulosa* | 94 | 97 |
|  |  | Longxi-hongkou | 19930424 | *D. involucrate* | 425 | 456 |
|  |  | Cuiyunlanggubai | 20000301 | *P. orientalis* | 520 | 608 |
|  |  | Guangwushan | 19920201 | Forest ecosyatem | - | 265 |
|  |  | Zhuchangou | 20010608 | Forest ecosystem | - | 187 |
|  |  | Minjiangbai | 20010608 | *C. chengiana* | - | 67 |
| *A. accanala* | Guangdong | Fengxi | 19840404 | Forest ecosystem | 92 | 92 |
|  |  | Heisidingkuoyelin | 19790801 | Subtropical evergreen broad-leaved forest | 95 | 95 |
|  |  | Qimuzhang | 19900701 | Subtropical evergreen broad-leaved forest, *A. spinulosa* | - | 84 |
|  | Yunnan | Amushan | 19951201 | Forest ecosystem | 81 | 81 |
|  |  | Wenshanlaojunshan | 20031004 | Monsoonal evergreen broad-leaved forest | 99 | 178 |
|  |  | Dazhongshan | 20030401 | *T. sinense* | - | 203 |
|  | Guizhou | Duyi | 19900501 | Subtropical ravine rain forest | - | 74 |
|  | Sichuan | Panzhihuasutie | 19830101 | *C. panzhihuaensis*: | - | 164 |
| *R. communis* | Yunnan | Gaoligongshan | 19830101 | Forest ecosystem | 178 | 162 |
|  |  | Guanyinshan | 19940527 | Subtropical evergreen broad-leaved forest | - | 79 |
|  | Guizhou | Chishuisuoluo | 19840911 | *A. spinulosa* | 104 | 84 |
|  | Hubei | Mulinzi | 19830101 | Mid-subtropical forest ecosystem | 87 | 92 |
|  |  | Houhe | 19850101 | Mid-subtropical forest ecosystem | 99 | 236 |
|  |  | Shennongjia | 19860709 | Mid-subtropical forest ecosystem, *D. involucrate* | 96 | 101 |
|  |  | Jiugongshan | 19810201 | *P. amabili, F. longipetiolata*, *L. chinense, C. japonicum* | 94 | 86 |
|  |  | Daguisi | 20020525 | North subtropical forest ecosystem | - | 42 |
|  | Gansu | Xiangshan | 19920703 | forest ecosystem | 132 | 213 |
|  | Shandong | Yuanshan | 19860826 | *P. orientalis, P. tabuliformis* | 86 | 93 |
|  |  | Culaishan | 19991101 | Warm temperate broad-leaved forest | - | 76 |
|  | Henan | Liankangshan | 19820624 | Mixed Eevergreen and Deciduous Broadleaved Forest | 184 | 168 |
|  |  | Jingangtai | 19820624 | Transitional areas forest ecosystem | 54 | 87 |
|  |  | Jigongshan | 19820624 | forest ecosystem | - | 73 |
|  | Hunan | Bamianshan | 19820409 | Forest ecosystem, *C. argyrophylla* | 144 | 159 |
|  |  | Liuyangdaweishan | 19820409 | forest ecosystem | - | 85 |
|  |  | Xinningshunhuangshan | 19820401 | Subtropical evergreen broad-leaved forest, *C. argyrophylla, A. fabri* | - | 81 |
|  | Jiangxi | Jinggangshan | 19810301 | Subtropical evergreen broad-leaved forest | - | 76 |
|  | Zhejiang | Fengyangshan-baishanzu | 19750101 | *A. beshanzuensi* | - | 77 |

The full name of species: *T. chinensis*: *Taxus chinensis* (Pilger) Rehd.; *B. sinensis*: *Bretschneidera sinensis*; *G. biloba*: *Ginkgo biloba* L.; *A. spinulosa*: *Alsophila spinulosa* (Wall. ex Hook.) R.M. Tryon; *D. involucrate*: *Davidia involucrate* Baill.; *P. orientalis*: *Platycladus orientalis* (L.) Franco; *F. longipetiolata*: *Fagus longipetiolata* Seem.; *C. chengiana*:*Cupressus chengiana* S. Y. Hu; *T. sinense*: *Tetracentron sinense* Oliv.; *C. panzhihuaensis*: *Cycas panzhihuaensis* L. Zhou et S. Y. Yang; *L. chinense*: *Liriodendron chinense* (Hemsl.) Sargent.; *P. amabilis*: *Pseudolarix amabilis* (Nelson) Rehd.; *C. japonicum*: *Cercidiphyllum japonicum* Sieb. et Zucc.; *C. argyrophylla*: *Cathaya argyrophylla* Chun et Kuang; *A. fabri*: *Abies fabri* (Mast.) Craib; *A. beshanzuensis*: *Abies beshanzuensis* M. H. Wu; *P. tabuliformis*: *Pinus tabuliformis* Carr.

**Table S2. The list of 154 woody oil plants**

| **Family** | **Genus** | **Species** |
| --- | --- | --- |
| Aceraceae | Acer | *Acer truncatum* Bunge |
| Anacardiaceae | Dracontomelon | *Dracontomelon duperreanum* Pierre |
|  | Dracontomelon | *Dracontomelon macrocarpum* H. L. Li |
|  | Pistacia | *Pistacia chinensis* Bunge |
|  | Pistacia | *Pistacia vera* L. |
|  | Anacardium | *Anacardium occidentale* L. |
|  | Rhus | *Rhus chinensis* Mill. |
| Apocynaceae | [Nerium](http://frps.eflora.cn/frps/Nerium) | *Nerium indicum* Mill. |
|  | Cerbera | *Cerbera manghas* L. |
| Betulaceae | Corylus | *Corylus heterophylla* Fisch. |
|  | Corylus | *Corylus ferox* Wall. |
|  | Corylus | *Corylus yunnanensis* A. Camus |
|  | Corylus | *Corylus ferox* Wall. var. thibetica (Batal.) Franch. |
|  | Corylus | *Corylusmandshurica* Maxim. |
| Boraginaceae | Cordia | *Cordiadichotoma* Forst. f. |
| Burseraceae | Canarium | *Canarium album* (Lour.) Raeusch. |
|  | Canarium | *Canarium pimela* Leenh. |
|  | Canarium | *Canarium bengalense* Roxb. |
| Calycanthaceae | Chimonanthus | *Chimonanthus nitens* Oliv. |
| Caprifoliaceae | Sambucus | *Sambucus williamsii* Hance |
| Celastraceae | Celastrus | *Celastrus angulatus* Maxim. |
|  | Celastrus | *Celastrus paniculatus* Willd. |
|  | Celastrus | *Celastrus hindsii* Benth. |
|  | Celastrus | *Celastrus angulatus* Maxim. |
|  | Celastrus | *Celastrus orbiculatus* Thunb. |
|  | Euonymus | *Euonymus alatus* (Thunb.) Sieb. |
|  | Euonymus | *Euonymus hamiltonianus* Wall. ex Roxb. |
|  | Euonymus | *Euonymus grandiflorus* Wall. |
|  | Euonymus | *Euonymus acanthocarpus* Franch. |
|  | Euonymus | *Euonymus myrianthus* Hemsl. |
|  | Euonymus | *Euonymus maackii* Rupr. |
|  | Euonymus | *Euonymus carnosus* Hemsl. |
|  | Euonymus | *Euonymus japonicas* Thunb. |
|  | Euonymus | *Euonymus tingens* Wall. |
| Cephalotaxaceae | Cephalotaxus | *Cephalotaxus fortune* Hook. f. |
|  | [Cephalotaxus](http://frps.eflora.cn/frps/Cephalotaxaceae) | *Cephalotaxus sinensis* (Rehd. et Wils. ) Li |
| Combretaceae | Terminalia | *Terminalia catappa* L. |
| Cornaceae | Swida | *Swida wilsoniana* (Wanger.) Sojak |
|  | Swida | *Swida walteri* (Wanger.) Sojak |

Continue table S2

| **Family** | **Genus** | **Species** |
| --- | --- | --- |
| Cucurbitaceae | Hodgsonia | *Hodgsonia macrocarpa* (Bl.) Cogn. |
| Elaeocarpaceae | Sloanea | *Sloanea hemsleyana* (Ito) Rehd. et Wils. |
| Euphorbiaceae | Aleurites | *Aleurites moluccana* (L.) Willd. |
|  | Deutzianthus | *Deutzianthus tonkinensis* Gagnep. |
|  | Hevea | *Hevea brasiliensis* (Willd. ex A. Juss.) Muell. Arg. |
|  | Jatropha | *Jatropha curcas* L. |
|  | Lasiococca | *Lasiococca comberi* Haines var. pseudoverticillata (Merr.) H. S. Kiu |
|  | Macaranga | *Macaranga adenantha* Gagnep. |
|  | Ricinus | *Ricinus communis* L. |
|  | Sapium | *Sapium sebiferum* (L.) Roxb. |
|  | Sapium | *Sapium discolor* (Champ. ex Benth.) Muell. Arg. |
|  | Vernicia | *Vernicia fordii* (Hemsl.) Airy Shaw |
|  | Vernicia | *Vernicia montana* Lour. |
| Fagaceae | Fagus | *Fagus longipetiolata* Seem. |
| Flacourtiaceae | Hydnocarpus | *Hydnocarpus kurzii* (King) Warb. |
|  | Hydnocarpus | *Hydnocarpus hainanensis* |
|  | Idesia | *Idesia polycarpa* Maxim. |
|  | Idesia | *Idesia polycarpa* Maxim. var. vestita Diels |
| Guttiferae | Garcinia | *Garcinia multiflora* Champ. ex Benth. |
|  | Garcinia | *Garcinia oblongifolia* Champ. ex Benth. |
|  | Mesua | *Mesua ferrea* L. |
| Hamamelidaceae | Corylopsis | *Corylopsis multiflora* Hance |
| Juglandaceae | Juglans | *Juglans mandshurica* |
|  | Juglans | *Juglans regia* |
|  | Juglans | *Juglans sigillata* |
|  | Juglans | *Juglans cathayens*is |
|  | Carya | *Carya illinoensis* (Wangenh.) K. Koch, Dendr. |
|  | Carya | *Carya cathayensis* |
| Lauraceae | Cinnamomum | *Cinnamomum burmanni* (Nees et T.Nees) Blume |
|  | Cinnamomum | *Cinnamomum camphora* (L.) presl |
|  | Cinnamomum | *Cinnamomum porrectum* (Roxb.) Kosterm. |
|  | Cinnamomum | *Cinnamomum japonicum* Sieb. |
|  | Cinnamomum | *Cinnamomum glanduliferum* (Wall.) Nees |
|  | Cinnamomum | *Cinnamomum saxatile* H. W. Li |
|  | Cinnamomum | *Cinnamomum longepaniculatum* (Gamble) N. Chao ex H. W. Li |
|  | Lindera | *Lindera glauca* (Sieb. et Zucc.) Bl |
|  | Lindera | *Lindera megaphylla* Hemsl. |
|  | Lindera | *Lindera communis* Hemsl. |
|  | Lindera | *Lindera caudate* (Nees) Hook. f. |
|  | Lindera | *Lindera latifolia* Hook. f. |
|  | Lindera | *Lindera nacusua* (D. Don) Merr. |

Continue table S2

| **Family** | **Genus** | **Species** |
| --- | --- | --- |
| Lauraceae | Lindera | *Lindera metcalfiana* Allen |
|  | Lindera | *Lindera reflexa* Hemsl. |
|  | Lindera | *Lindera aggregate* (Sims) Kosterm |
|  | Litsea | *Litsea cubeba* (Lour.) Pers. |
|  | Litsea | *Litsea pungens* Hemsl. |
|  | Neolitsea | *Neolitsea aurata* (Hay.) Koidz. |
|  | Neolitsea | *Neolitsea chuii* Merr. |
|  | Neocinnamomum | *Neocinnamomum delavayi* (Lec.) Liou |
|  | Neocinnamomum | *Neocinnamomum caudatum* (Nees) Merr. |
| Magnoliaceae | Magnolia | Magnolia denudate Desr. |
|  | Magnolia | *Magnolia grandiflora* L. |
|  | Michelia | *Michelia maudiae* Dunn |
|  | Michelia | *Michelia chapensis* Dandy |
|  | Michelia | *Michelia sphaerantha* C. Y. Wu |
|  | Michelia | *Michelia hedyosperma* Law |
|  | Tsoongiodendron | *Tsoongiodendron odorum* |
|  | [Illicium](http://frps.eflora.cn/frps/Illicium) | *Illicium verum* |
|  | Schisandra | *Schisandra chinensis* (Turcz.) Baill. Hist. Pl. |
|  | Schisandra | *Schisandra sphenanthera* |
|  | Schisandra | *Kadsura longipedunculata* |
| Meliaceae | Melia | *Melia azedarach* L. |
|  | Melia | *Melia toosendan* Sieb. et Zucc |
|  | Melia | *Aphanamixis polystachya* (Wall.) R. N. Parker |
| Myristicaceae | Horsfieldia | *Horsfieldia pandurifolia* Hu |
| Olacaceae | Malania | *Malania oleifera* Chun et S. Lee ex S. Lee |
| Oleaceae | Olea | *Olea europaea* L. |
| Palmae | Cocos | *Cocos nucifera* L. |
|  | Elaeis | *Elaeis guineensis* Jacq. |
| Pinaceae | Keteleeria | *Keteleeria davidiana* (Bertr.) Beissn. |
|  | Keteleeria | *Keteleeria evelyniana* Mast. |
|  | Picea | *Picea asperata* Mast. |
|  | Picea | *Picea koraiensis* Nakai |
|  | Pinus | *Pinus densiflora* Sieb. et Zucc. |
|  | Pinus | *Pinus kwangtungensis* Chun ex Tsiang |
|  | Pinus | *Pinus tabuliformis* Carr. |
|  | Pinus | *Pinus thunbergii* Parl. |
|  | Pinus | *Pinus armandii* Franch. |
|  | Pinus | *Pinus bungeana* Zucc. ex Endl. |
|  | Pinus | *Pinus koraiensis* Sieb. et Zucc. |
|  | Pinus | *Pinus massoniana* Lamb. |

Continue table S2

| **Family** | **Genus** | **Species** |
| --- | --- | --- |
| Pinaceae | Pinus | *Pinus yunnanensis* Franch. |
|  | Larix | *Larix olgensis* Henry |
|  | Larix | *Larix gmelinii* (Rupr.) Kuzen. |
| Rosaceae | Armeniaca | *Armeniaca vulgaris* Lam. |
|  | Armeniaca | *Armeniaca sibirica* (L.) Lam. |
|  | Amygdalus | *Amygdalus davidiana* (Carrière) de Vos ex Henry |
|  | Amygdalus | *Amygdalus communis* L. |
|  | Laurocerasus | *Laurocerasus undulate* (D. Don) Rocm. |
| Rutaceae | Citrus | *Citrus maxima* (Burm.) Merr. |
|  | Citrus | *Citrus limon* (L.) Burm. f. |
| Santalaceae | Pyrularia | *Pyrularia edulis* (Wall.) A. DC. Prodr. |
|  | Scleropyrum | *Scleropyrum wallichianum* |
| Sapindaceae | Amesiodendron | *Amesiodendron chinense* (Merr.) Hu |
|  | Delavaya | *Delavaya toxocarpa* Franch. |
|  | Koelreuteria | *Koelreuteria bipinnata* Franch. |
|  | Sapindus | *Sapindus mukorossi* Gaertn. |
|  | Xanthoceras | *Xanthoceras sorbifolium* Bunge |
| Sapotaceae | Madhuca | *Madhuca pasquieri* (Dubard) Lam. |
|  | Butyrospermum | *Butyrospermum parkii* Kotschy |
|  | Eberhardtia | *Eberhardtia aurata* (Pierre ex Dubard) Lec*.* |
|  | Eberhardtia | *Eberhardtia tonkinensis* Lec. |
| Styracaceae | Melliodendron | *Melliodendron xylocarpum* Hand.-Mazz. |
| Simaroubaceae | Picrasma | *Picrasma quassioides* (D. Don) Benn. |
|  | Picrasma | *Picrasma chinensis* P. Y. Chen |
| Taxaceae | Torreya | *Torreya grandis* Fort. et Lindl. cv. Merrillii |
| Theaceae | Camellia | *Camellia oleifera* Abel. |
|  | Camellia | *Camellia japonica* L. |
|  | Camellia | *Camellia reticulate* Lindl. |
|  | Camellia | *Camellia pitardii* Coh. St. var. yunnanica Sealy |
|  | Camellia | *Camellia chekiangoleosa* Hu |
|  | Camellia | *Camellia oleifera* Abel. var. monosperma Chang |
|  | Camellia | *Camellia semiserrata* Chi |
| Ulmaceae | Celtis | *Celtis philippensis* Blanco var. philippensis |
| Proteaceae | Macadamia | *Macadamia ternifolia* F. Muell. |

**Table S3.** **General circulation models (GCMs) used in this study**

| **GCMs** | **Code** | **Modeling Center (or Group)** |
| --- | --- | --- |
| ACCESS1-0 | AC | Commonwealth Scientific and Industrial Research Organization (CSIRO) and Bureau of Meteorology (BOM), Australia |
| BCC-CSM1-1 | BC | Beijing Climate Center, China Meteorological Administration |
| CCSM4 | CC | National Center for Atmospheric Research |
| CNRM-CM5 | CN | Centre National de Recherches Météorologiques / Centre Européen de Recherche et Formation Avancée en Calcul Scientifique |
| GFDL-ESM2G | GD | NOAA Geophysical Fluid Dynamics Laboratory |
| GFDL-CM3 | GF | NOAA Geophysical Fluid Dynamics Laboratory |
| GISS-E2-R | GS | NASA Goddard Institute for Space Studies |
| HadGEM2-AO | HD | National Institute of Meteorological Research/Korea Meteorological Administration |
| HadGEM2-ES | HE | Met Office Hadley Centre (additional HadGEM2-ES realizations contributed by Instituto Nacional de Pesquisas Espaciais) |
| HadGEM2-CC | HG |
| INMCM4 | IN | Institute for Numerical Mathematics |
| IPSL-CM5A-LR | IP | Institut Pierre-Simon Laplace |
| MIROC5 | MC | Atmosphere and Ocean Research Institute (The University of Tokyo), National Institute for Environmental Studies, and Japan Agency for Marine-Earth Science and Technology |
| MRI-CGCM3 | MG | Meteorological Research Institute |
| MIROC-ESM-CHEM | MI | Japan Agency for Marine-Earth Science and Technology, Atmosphere and Ocean Research Institute (The University of Tokyo), and National Institute for Environmental Studies |
| MIROC-ESM | MR |
| MPI-ESM-LR | MP | Max-Planck-Institut für Meteorologie (Max Planck Institute for Meteorology) |
| NorESM1-M | NO | Norwegian Climate Centre |
